# Efficacy of gut microbiota-targeted therapies in Parkinson’s disease: a systematic review and meta-analysis of randomized controlled trials

**DOI:** 10.3389/fcimb.2025.1627406

**Published:** 2025-09-25

**Authors:** Xiang Gu, Jihong Tang, Chanxi Chen

**Affiliations:** ^1^ Integrated Diagnosis and Treatment Center for Neurological Diseases, Yubei District People’s Hospital of Chongqing, Chongqing, China; ^2^ Department of Respiratory and Critical Care Medicine, Yubei District People’s Hospital of Chongqing, Chongqing, China

**Keywords:** meta-analysis, Parkinson’s disease, probiotics, synbiotics, antibiotics, fecal microbiota transplantation

## Abstract

**Objective:**

This study aimed to investigate the efficacy of gut microbiota (GM)-targeted therapies in treating Parkinson’s disease (PD).

**Methods:**

Randomized controlled trials (RCTs) were retrieved from PubMed, Embase, Cochrane, and WOS from database inception to June 2025. The eligible RCTs employed GM-targeted therapies, including antibiotics, probiotics, synbiotics, or fecal microbiota transplantation (FMT), as adjunct treatments for PD. Data were pooled using a random-effects model, and the effect sizes were expressed as standardized mean differences (SMDs). In addition, the quality of evidence for all outcomes was assessed using the GRADE framework.

**Results:**

This study demonstrated that GM-targeted therapies significantly improved PD outcomes, including Movement Disorder Society-Unified Parkinson Disease Rating Scale (MDS-UPDRS) III (SMD: -0.34, 95%CI: -0.57 to -0.11, P = 0.004), bowel movements (BMs) (SMD: 1.27, 95%CI: 0.35 to 2.2), use of laxatives (SMD: -0.33, 95% CI: -0.65 to -0.02), malondialdehyde (MDA) (SMD: -0.69, 95%CI: -1.23 to -0.15) indicators. However, there were no significant improvements in MDS-UPDRS I (SMD: -0.64, 95%CI: -1.42 to 0.13), MDS-UPDRS II (SMD: -0.28, 95%CI: -0.70 to 0.14), MDS-UPDRS IV (SMD: -0.08, 95% CI: -0.82 to 0.66), Mini-Mental State Examination (MMSE) (SMD: -0.01, 95% CI: -0.30 to 0.29), Montreal Cognitive Assessment (MoCA) (SMD: 0.04, 95%CI: -0.53 to 0.60), non-motor symptom scale (NMSS) (SMD: -0.11, 95%CI: -0.94 to 0.72), Parkinson’s Disease Questionnaire-39 (PDQ-39) (SMD: -0.19, 95%CI: -0.58 to 0.20), total antioxidant capacity (TAC) (SMD: 0.29, 95%CI: -0.04 to 0.62), glutathione (GSH) (SMD: 0.51, 95%CI: -0.02 to 1.03), and Geriatric Depression Scale-15 (GDS-15) (SMD: -0.37, 95%CI: -0.87 to 0.12).

**Conclusion:**

GM-targeted therapies may improve motor symptom scores (as measured by MDS-UPDRS III), alleviate constipation, and reduce blood malondialdehyde levels in PD patients. However, they did not significantly impact the scores for cognitive function, PD neuropsychiatric, behavioral, and emotional symptoms, and activities of daily living in this analysis. Given the inherent limitations of the included studies (such as small sample sizes and heterogeneity), future large-scale and rigorously designed RCTs are needed to validate these preliminary findings.

**Systematic review registration:**

https://www.crd.york.ac.uk/prospero/, identifier CRD42024606415.

## 1 Introduction

Parkinson’s disease (PD), a neurodegenerative illness, is increasingly prevalent across the globe, severely affecting patients’ lives and causing huge economic strains on society. The primary hallmark of PD is the gradual loss of dopaminergic neurons (DNs) in the substantia nigra (SN). This neuronal death leads to classical motor symptoms, including resting tremors, bradykinesia, and muscle rigidity. Various non-motor symptoms affect PD patients, including anxiety, depression, constipation, sleep disturbances, and hyposmia. These non-motor symptoms often occur years before motor symptoms and severely impact PD patients’ daily functioning and quality of life (Lang, 2011). As the global population continues to age, the number of PD patients is projected to exceed 12 million by 2050 (Rocca, 2018). Although the exact causes of PD remain unclear, genetic susceptibility, environmental exposure, oxidative stress (OS), neuroinflammation, and gut microbiota dysbiosis (GMD) are believed to be strongly linked to the pathogenesis of PD (Simon et al., 2020).

In recent years, there has been growing attention on the influence of gut microbiota (GM) on the development and progression of PD. According to the gut-brain axis theory, GM communicates bidirectionally with the central nervous system through neural, endocrine, immune, and metabolic pathways (Cryan et al., 2019).Alterations in the abundance of certain bacterial taxa, including a common observation of reduced levels of bacteria like lactobacilli and bifidobacteria (often associated with health benefits in some contexts) and an increase in others such as Enterobacteriaceae and Prevotella species (often linked to pro-inflammatory conditions). However, it is crucial to note that these classifications are not absolute in the context of PD (Hill-Burns et al., 2017). This dysbiosis can lead to PD progression via gut barrier disruption, chronic inflammation, and the disruption of the central nervous system resulting from the imbalanced metabolism of short-chain fatty acids (SCFAs) (Poewe et al., 2017). Holmqvist et al. provided direct evidence in rats that α-synuclein, a key pathological protein in PD, can be transmitted from the gut to the brain via the vagus nerve, suggesting a potential role of the gut in the initiation and progression of PD pathology (Holmqvist et al., 2014). Increasing evidence indicates that neurochemicals and metabolites, which may directly or indirectly impact the body’s physiological processes and the onset and progression of PD, can be synthesized and regulated by GM (Jameson et al., 2020). GM and metabolites in PD patients differ from those of healthy individuals, with more pro-inflammatory cytokines and fewer anti-inflammatory cytokines (Nuzum et al., 2020). A study suggested that GM may correlate with the severity of PD symptoms (Scheperjans et al., 2015).

GM-targeted therapies have become a research focus in recent years. Probiotics, prebiotics, and fecal microbiota transplantation (FMT) are considered to be able to improve PD symptoms. Animal studies showed that FMT can alleviate motor symptoms and reduce neuroinflammation and neuronal loss in PD mouse models (Sun et al., 2018). In PD mice, FMT can mitigate GM alterations and reduce inflammation by activating microglial and astrocytic cells in the SN. FMT may work by modulating GM balance, lowering OS, enhancing anti-inflammatory responses, and improving gut-brain axis function (Tamtaji et al., 2019). Nurrahma et al. (2021) demonstrated that probiotic supplementation in PD mice significantly improved their motor function and increased antioxidant enzyme activities (Tsao et al., 2021). Tan et al. found that in PD patients, probiotics can effectively alleviate such non-motor symptoms as constipation and anxiety (Tan et al., 2021). However, whether GM-targeted treatments can effectively improve PD symptoms and their safety remains unestablished. This meta-analysis seeks to appraise the efficacy and safety of GM-targeted therapies in PD, providing references for clinical trials and theoretical support for future applications of these therapies in treating PD.

## 2 Materials and methods

### 2.1 Protocol and registration

This study was registered in the International Prospective Register of Systematic Reviews (PROSPERO) under the number CRD42024606415. Results were reported based on the Preferred Reporting Items for Systematic Reviews and Meta-Analyses (PRISMA), PRISMA-extension statement, and PRISMA protocols (Hutton et al., 2015).

### 2.2 Literature search

PubMed, Embase, Cochrane, and WOS were retrieved for pertinent randomized controlled trials (RCTs) published from database inception to June 2025. The RCTs on the efficacy and safety of GM-targeted therapies in PD were included. The search terms included “RCT,” “clinical trial” clinical study,” “intervention study,” “Probiotics,” “Synbiotics,” “Antibiotic,” and “fecal transplantation.” Animal studies were excluded.

### 2.3 Inclusion and exclusion criteria

Inclusion Criteria:

P: Patients diagnosed with PD
I: GM-targeted therapies, including probiotics, synbiotics, fecal transplantation, and antibiotics
C: Placebo or conventional treatment
O: Primary outcomes: Clinical effectiveness, adverse reaction rate
Secondary outcomes: Movement Disorder Society-Unified Parkinson Disease Rating Scale (MDS-UPDRS) I, II, III, IV; Montreal Cognitive Assessment (MoCA); Parkinson’s Disease Questionnaire-39 (PDQ-39); Non-Motor Symptom Scale (NMSS); Mini-Mental State Examination (MMSE); Bowel movements (BMs); Geriatric Depression Scale-15 (GDS-15)
S: RCT

Exclusion Criteria:

(1) Letters, case reports, meeting abstracts, comments, and reviews; (2) Literature lacking insufficient data to calculate Standardized Mean Difference (SMD) or Relative Risk (RR) and 95% Confidence Interval (CI); (3) Studies lacking efficacy and/or safety data; (4) Studies with duplicated or overlapping data.

### 2.4 Data extraction

Data were gathered independently by two researchers. Any disagreements were addressed by a third investigator. The data extracted were as follows: first author, age, study duration, study design, year of publication, country, sample size, body mass index (BMI), MDS-UPDRS I, II, III, IV, NMSS, MMSE, MoCA, PDQ-39, BMs, glutathione (GSH), use of laxatives, malondialdehyde (MDA), total antioxidant capacity (TAC), and GDS-15. When continuous variables were expressed as medians with ranges or interquartile ranges, the mean ± standard deviation was computed via a reliable mathematical method (Luo et al., 2018).

### 2.5 Quality assessment

Two researchers independently appraised the eligible RCTs via the Cochrane risk-of-bias (RoB) tool, and then cross-verified their evaluations. Seven RoB items were evaluated: incomplete outcome data, blinding of participants and personnel, allocation concealment, selective reporting, random sequence generation, and other biases. Studies were classified into three categories based on their methodological quality: “high risk of bias,” “low risk of bias,” and “unclear bias.” Any disagreements were resolved by a third researcher.

### 2.6 Statistical analysis

Review Manager 5.4.1 was employed to conduct the statistical analysis. Continuous data were expressed as weighted mean difference (WMD) or SMD; dichotomous data were reported as RR. Each indicator was reported with 95% CIs using a random-effects model. The Chi-squared (χ2) test and inconsistency index (I^2^) were employed to assess the heterogeneity of the results (Higgins and Thompson, 2002). P value < 0.1 or I^2^ > 50% indicated high heterogeneity. The publication bias was assessed by Egger’s regression tests (Egger et al., 1997) and performed by Stata 15.1 (Stata Corp, College Station, Texas, USA). P < 0.05 suggested statistically significant publication bias. Additionally, the quality of evidence for each outcome was graded as “high”, “moderate”, “low”, or “very low” under Grading of Recommendations, Assessment, Development, and Evaluations (GRADE) (Guyatt et al., 2011).

## 3 Results

### 3.1 Retrieval results

**Figure 1:**
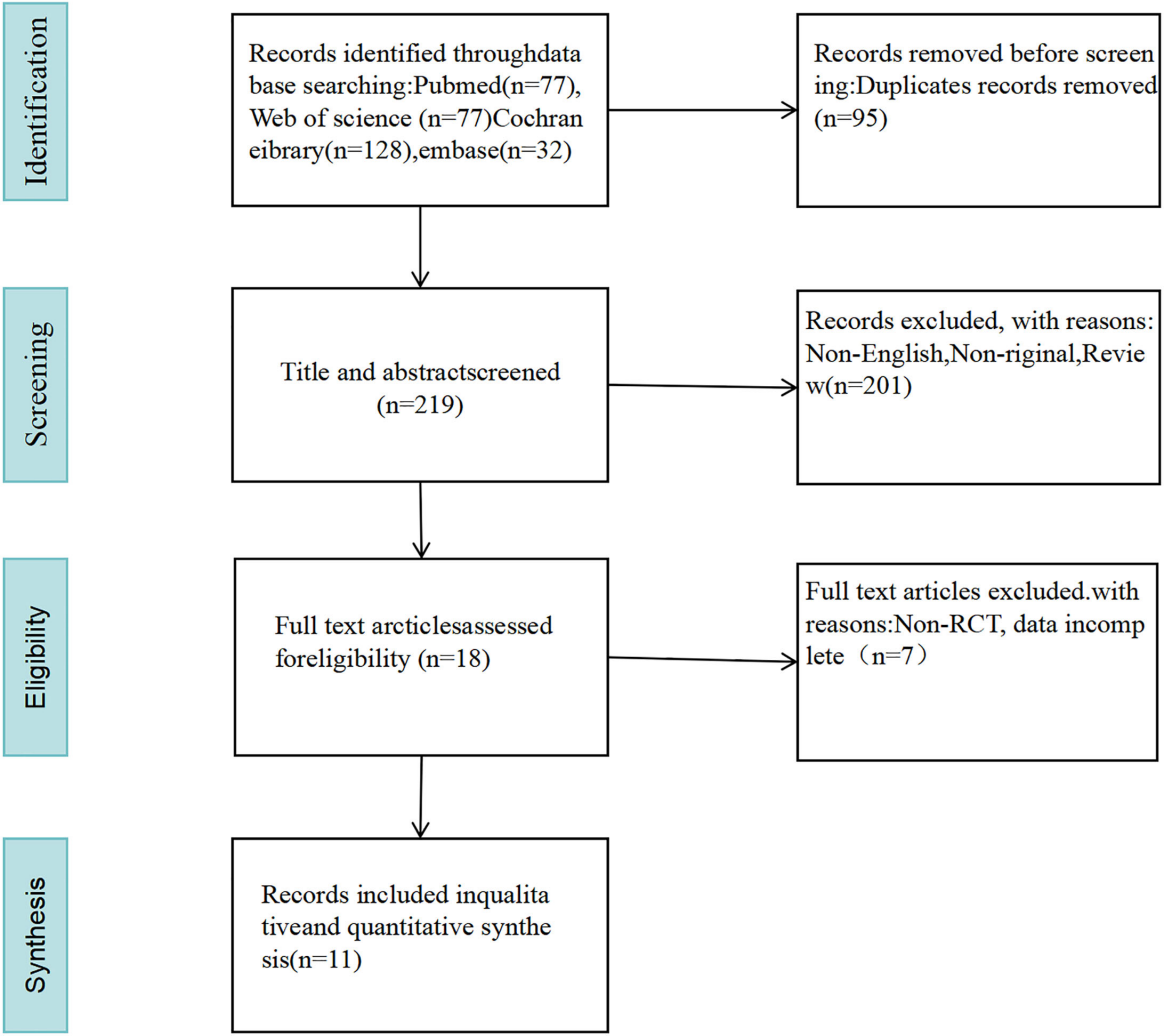
Flow plot.

A total of 314 pertinent studies were selected from the databases. After eliminating 95 duplicates, 289 studies were assessed by reviewing their titles and abstracts. A total of 176 studies were excluded for being non-original, non-English, and reviews. Ultimately, 11 studies were included after a full-text reading (Barichella et al., 2016; Tamtaji et al., 2019; Ibrahim et al., 2020; Tan et al., 2021; Cheng et al., 2023; DuPont et al., 2023; Ghalandari et al., 2023; Mehrabani et al., 2023; Yang et al., 2023; Scheperjans et al., 2024; Ramadan et al., 2025) (as illustrated in **Figure 1**).

### 3.2 Characteristics of included studies

Eleven studies with 711 patients were included in this study. A total of 381 individuals were in the intervention group and 330 were in the control group. The sample sizes ranged from a minimum of 11 to a maximum of 128 participants. The treatment duration varied between 4 weeks and 12 months. The intervention group received probiotics, such as Lactobacillus acidophilus, Bifidobacterium bifidum, Lactobacillus reuteri, and Lactobacillus fermentum, as well as fermented milk containing Lacticaseibacillus paracasei strain Shirota (LcS), FMT, and capsules containing Lactobacillus bulgaricus, Lactobacillus plantarum, Lactobacillus acidophilus, Lactobacillus casei, Bifidobacterium longum, Bifidobacterium breve, Bifidobacterium infantis, and Streptococcus thermophilus (each genus containing 1.5×10¹¹ CFU). The control group received placebo capsules, which contained maltodextrin and starch. The demographics and clinical characteristics of the selected studies are illustrated in **Table 1**.

**Table 1:** Demographics and clinical characteristics.

Author	Study period	Country	Study design	Patients (Intervention/Placebo)		Follow-up	Age (Intervention/Placebo)		BMI (Intervention/Placebo)		Disease duration (years) (Intervention/Placebo)		Sex Male/Female (Intervention/Placebo)	
Tamtaji 2018 (Tamtaji et al., 2019)	2017,8-2017,12	Iran	RCT	30	30	12-week	68.2 ± 7.8	67.7 ± 10.2	26.6 ± 3.2	25.8 ± 3.5				
XiaodongYang 2023 (Yang et al., 2023)	2018,8-2019,01	China	RCT	65	63	12-week	67.22 ± 6.46	69.64 ± 6.41	22.58 ± 3.17	22.94 ± 2.95				
Yi Cheng 2023 (Cheng et al., 2023)	2019,2-2019,12	China	RCT	27	27	12-week	60.52 ± 8.68	62.63 ± 8.41	22.33 ± 2.16	22.43 ± 2.16	6.29 ± 4.47	6.51 ± 4.92	15/12	17 /10
Ghalandari 2023 (Ghalandari et al., 2023)	2022,09-2023,01	Iran	RCT	13	14	8-week	68.07 ± 6.68	68.54 ± 6.92					7/6	8/6
DuPont 2023 (DuPont et al., 2023)	2019,6-2020,05	USA	RCT	7	4	12-month	68.26 ± 5.13	66.24 ± 8.58			2 ± 0.70	4.76 ± 33.81	5/2	4/0
Ibrahim 2020 (Ibrahim et al., 2020)	2018,10-2019,02	Malaysian	RCT	22	26	8-week	69.0 ± 7.83	70.96 ± 14.30	22.36 ± 3.96	23.05 ± 5.88	7.07 ± 3.91	7.14 ± 6.41	16/6	17/9
Barichella 2016 (Barichella et al., 2016)	2015,06-2015,10	Italyn	RCT	80	40	4-week	71.8 ± 7.7	69.5 ± 10.3	24.6 ± 5.9	24.7 ± 4.4	10.9 ± 6.7	9.6 ± 6.3	41/39	24/16
Tan 2020 (Tan et al., 2021)	2017,12-2019,01	Malaysian	RCT	34	38	4-week	70.9 ± 6.6	68.6 ± 6.7			9.7 ± 5.1	10.1 ± 7.6	20/14	28/10
Mehrabani 2023 (Mehrabani et al., 2023)	2021,05-2021,09	Iran	RCT	40	40	12-week	68.2 ± 7.68	69.05 ± 8.23			5.72 ± 2.86	5.28 ± 1.76	21/19	20/20
Filip Scheperjans 2024 (Scheperjans et al., 2024)	2020,11-2023,06	Finland	RCT	30	15	12-month	64.9 ± 8.17	62.27 ± 14.31	26.28 ± 5.12	25.93 ± 2.91	5.727± 2.88	6.0± 5.13	16/17	15/18
Mohamed E 2025 (Ramadan et al., 2025)	2022,08-2024,04	Egypt	RCT	33	33	3-month	55.7 ± 5.91	56.06 ± 6.2					16/17	15/18

RCT, Randomized Controlled Trial; BMI, Body Mass Index

### 3.3 Quality assessment results

**Figure 2:**
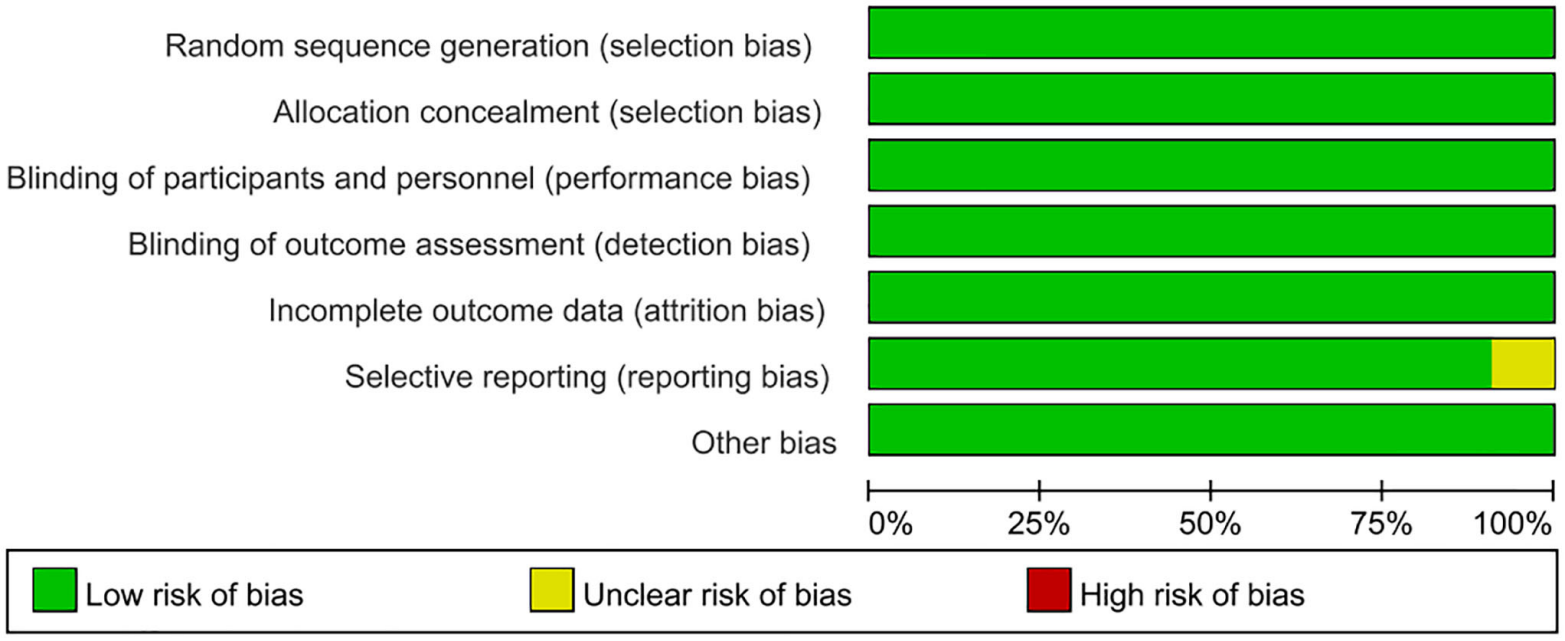
Risk of bias graph.

**Figure 3:**
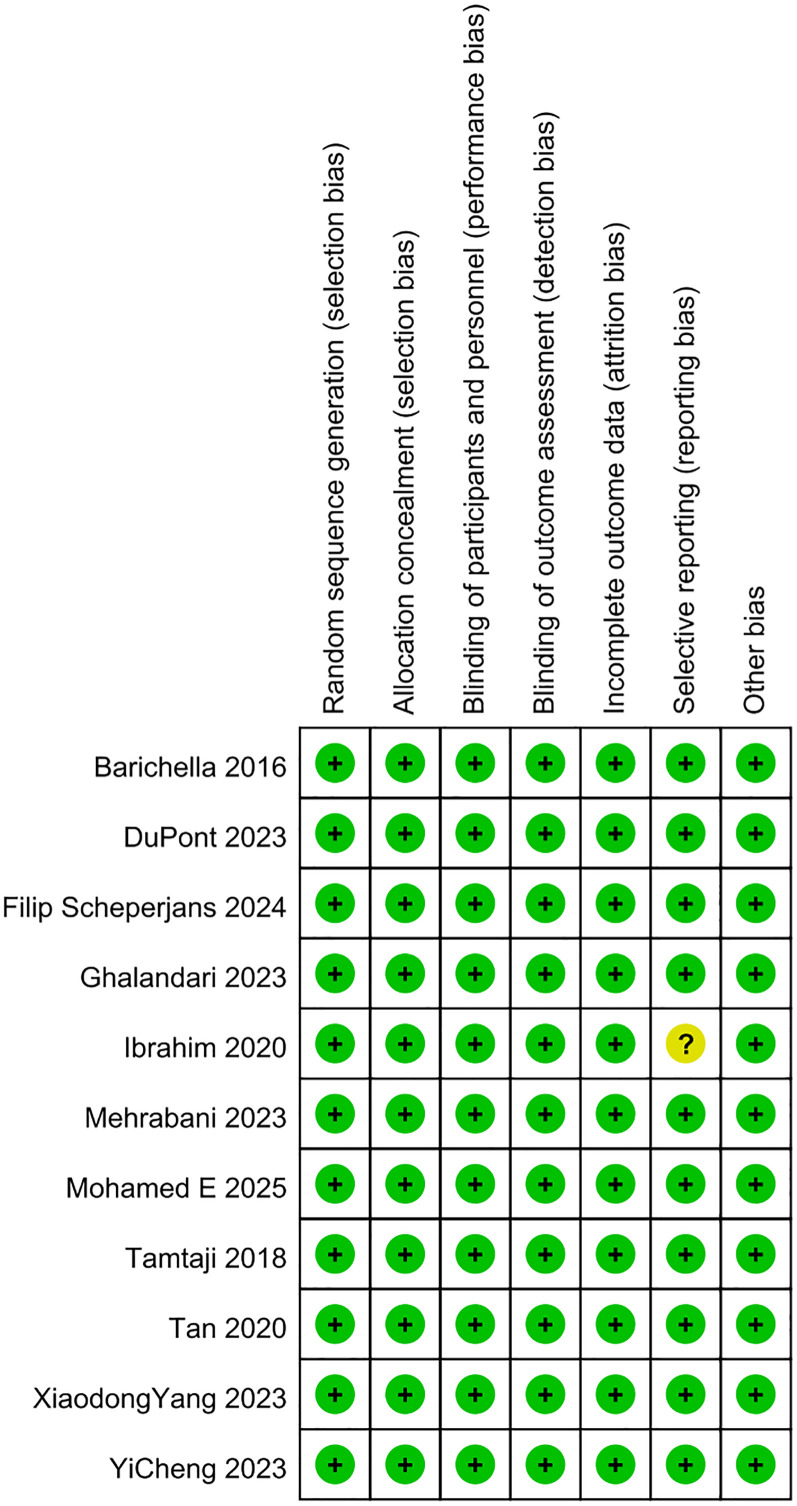
Risk of bias summary.

In this meta-analysis, the quality of the included studies was appraised via the Cochrane RoB tool. As illustrated in **Figures 2**, **3**, the risk of selection bias, performance bias, detection bias, attrition bias, and reporting bias of the nine eligible studies was evaluated as low, respectively. Regarding the selective reporting, this study (Ibrahim et al., 2020) was rated as unclear (Ibrahim et al., 2020).

### 3.4 Meta-analysis results

#### 3.4.1 Change in MDS-UPDRS I (Movement Disorders Society-Unified Parkinson's Disease Rating Scale)

**Figure 4:**
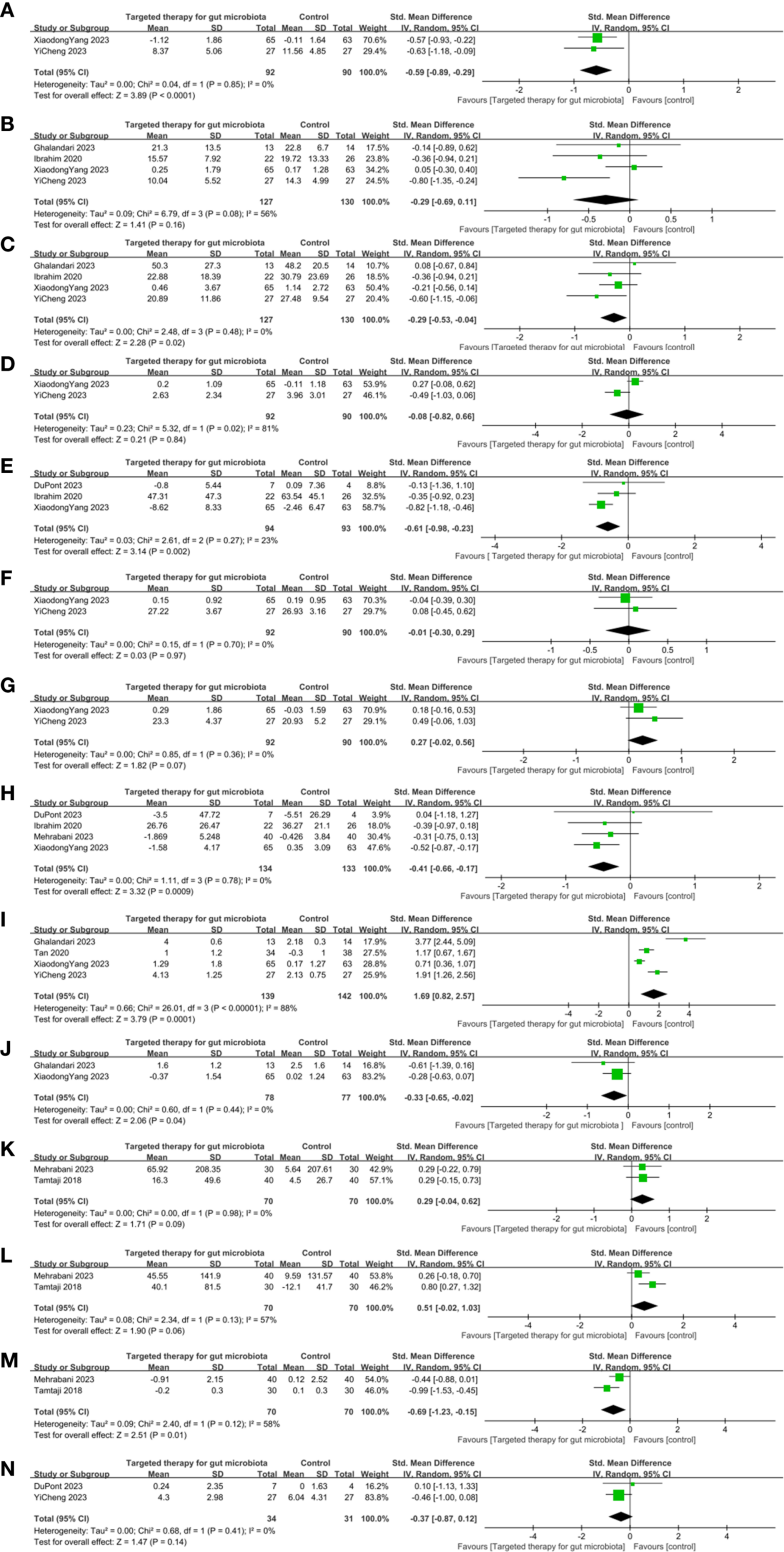
Effects of gut microbiota-targeted therapies on clinical and biochemical profiles in Parkinson’s disease. **(A)** MDS-UPDRS I Forest plot, **(B)** MDS-UPDRS II Forest plot, **(C)** MDS-UPDRS III Forest plot, **(D)** MDS-UPDRS IV Forest plot, **(E)** Non-motor symptom scale Forest plot, **(F)** MMSE Forest plot, **(G)** MoCA Forest plot, **(H)** PDQ-39 Forest plot, **(I)** BMsForest plot, **(J)** Use of laxatives Forest plot, **(K)** TAC Forest plot, **(L)** GSH Forest plot, **(M)** MDA Forest plot, **(N)** GDS-15 Forest plot. Note: Clinical outcomes: motor symptoms, constipation, anxiety, cognitive; Biochemical outcomes: blood MDA, TAC, GSH, GDS-15 levels.

Four RCTs on MDS-UPDRS I were included. The meta-analysis showed no significant difference in the change of MDS-UPDRS I scores between the GM-targeted therapy group and the control group (SMD: -0.64, 95% CI: -1.42 to 0.13, P = 0.1) (**Figure 4A**), indicating that GM-targeted therapy had no significant effect on MDS-UPDRS I in PD patients.

#### 3.4.2 Change in MDS-UPDRS II

Six RCTs on MDS-UPDRS II were included. The difference between the GM-targeted therapy group and the control group in MDS-UPDRS II was statistically significant (SMD: -0.28, 95%CI: -0.70 to 0.14, P = 0.19) (**Figure 4B**). Therefore, GM-targeted therapies could not notably influence MDS-UPDRS II in PD patients.

#### 3.4.3 Change in MDS-UPDRS III

Six RCTs on MDS-UPDRS III were included. The result showed that the decrease in MDS-UPDRS III in the GM-targeted therapy group was substantially greater (SMD: -0.34, 95%CI: -0.57 to -0.11, P = 0.004) (**Figure 4C**) in contrast to the control group. Thus, GM-targeted therapies can notably improve MDS-UPDRS III in PD patients.

#### 3.4.4 Change in MDS-UPDRS IV

Four RCTs on MDS-UPDRS IV were included. The difference between the GM-targeted therapy group and the control group in MDS-UPDRS IV was statistically significant (SMD: -0.08, 95% CI: -0.82 to 0.66, P = 0.84) (**Figure 4D**). Therefore, GM-targeted therapies could not notably influence MDS-UPDRS IV in PD patients.

#### 3.4.5 Change in Non-Motor Symptom Scale

Four RCTs on NMSS were included. The meta-analysis showed no significant difference in the change of NMSS scores between the GM-targeted therapy group and the control group (SMD: -0.11, 95% CI: -0.94 to 0.72, P = 0.79) (**Figure 4E**), indicating that GM-targeted therapy had no significant effect on NMSS in PD patients.

#### 3.4.6 Change in Mini-Mental State Examination

Two RCTs on MMSE were included. The difference between the GM-targeted therapy group and the control group in MMSE was statistically significant (SMD: -0.01, 95% CI: -0.30 to 0.29, P = 0.97) (**Figure 4F**). Thus, GM-targeted therapies could not notably impact cognitive function in PD patients.

#### 3.4.7 Change in Montreal Cognitive Assessment

Three RCTs on MoCA were included. The difference between the GM-targeted therapy group and the control group in Moca was statistically significant (SMD: 0.04, 95%CI: -0.53 to 0.60, P = 0.90) (**Figure 4G**). Thus, GM-targeted therapies could not notably impact cognitive function in PD patients.

#### 3.4.8 Change in Parkinson's Disease Questionnaire-39

Five RCTs on PDQ-39 were included. The meta-analysis showed no significant difference in the change of PDQ-39 scores between the GM-targeted therapy group and the control group (SMD: -0.19, 95% CI: -0.58 to 0.20, P = 0.34) (**Figure 4H**), indicating that GM-targeted therapy had no significant effect on improving PDQ-39 scores in PD patients.

#### 3.4.9 Change in bowel movements

Five RCTs on BMs were included. The result demonstrated that the increase in BMs in the GM-targeted therapy group was substantially greater (SMD: 1.27, 95%CI: 0.35 to 2.20, P = 0.007) (**Figure 4I**) in contrast to the control group. Thus, GM-targeted therapies can greatly improve BMs in PD patients.

#### 3.4.10 Change in use of laxatives

Two RCTs on the use of laxatives were included. The result demonstrated that the decrease in the use of laxatives in the GM-targeted therapy group was substantially greater (SMD: -0.33, 95% CI: -0.65 to -0.02, P = 0.04) (**Figure 4J**) in contrast to the control group, suggesting that GM-targeted therapies can reduce the frequency of laxative use in PD patients.

#### 3.4.11 Change in TAC (plasma total antioxidant capacity)

Two RCTs on TAC were included. The difference between the GM-targeted therapy group and the control group in TAC was statistically significant (SMD: 0.29, 95% CI: -0.04 TO 0.62, P = 0.09) (**Figure 4K**). Thus, GM-targeted therapies could not improve TAC in PD patients.

#### 3.4.12 Change in GSH (total glutathione)

Two RCTs on GSH were included. The difference between the GM-targeted therapy group and the control group in GSH was statistically significant (SMD: 0.51; 95% CI: -0.02, 1.03; P = 0.06) (**Figure 4L**). Thus, GM-targeted therapies could not improve GSH in PD patients.

#### 3.4.13 Change in MDA (malondialdehyde)

Two RCTs on MDA were included. The result showed that the decrease in MDA in the blood in the GM-targeted therapy group was substantially greater in contrast to the control group (SMD: -0.69; 95% CI: -1.23, -0.15; P = 0.01) (**Figure 4M**). Thus, GM-targeted therapies can reduce MDA levels in the blood of PD patients.

#### 3.4.14 Change in Geriatric Depression Scale-15

Two RCTs on GDS-15 were included. The difference between the GM-targeted therapy group and the control group in GDS-15 was statistically significant (SMD: -0.37; 95% CI: -0.87, 0.12; P = 0.14) (**Figure 4N**). Thus, GM-targeted therapies could not notably impact GDS-15 in PD patients.

### 3.5 Assessment of publication bias

Egger’s test was performed for MDS-UPDRS I, MDS-UPDRS II, MDS-UPDRS III, the Non-motor Symptom Scale, PDQ-39, and BMs. The results indicated no evidence of publication bias for MDS-UPDRS I (P = 1.0), MDS-UPDRS II (P = 0.747), MDS-UPDRS III (P = 0.861), the Non-motor Symptom Scale (P = 0.404), PDQ-39 (P = 0.298), or BMs (P = 0.388).

### 3.6 GRADE rating

As shown in **Table 2**, the quality of evidence for MDS-UPDRS II, MDS-UPDRS IV, MoCA, PDQ-39, GSH, and MDA outcomes (PSA response rate) was graded as low. The quality of evidence for MDS-UPDRS I, MDS-UPDRS III, NMSS, MMSE, BMs, use of laxatives, TAC, and GDS-15 outcomes (PSA response rate) was graded as moderate. Based on the results above, the evidence for the effects of probiotics on improving MDS-UPDRS III scores, BMs, and use of laxatives in PD patients was of moderate certainty. However, the reliability of the results for other outcomes was compromised by study heterogeneity and publication bias, which led to a certain degree of downgrading in the quality of evidence. Therefore, further studies are warranted to validate these findings.

**Table 2:** RADE rating of each outcome.

No. of studies	Outcomes	SMD	95%CI	The overall validity p-value	*I* ^2^; P value	Risk of bias	Inconsistency I2	Indirectness	Imprecision p<0.001	Publication bias	Plausible confounding	Magnitude of effect	Dose-response gradient	GRADE
4	Change in MDS-UPDRS I	-0.64	-1.42,0.13	Null	89%;P<0.00001	No serious risk	Serious inconsistency	No serious	No serious	Undetected	Would not	No	No	Moderate
											reduce effect			
6	Change in MDS-UPDRS II	-0.28	-0.70, 0.14	Null	73%; P = 0.002	No serious risk	Serious inconsistency	No serious	Serious	Undetected	Would not	No	No	Low
											reduce effect			
6	Change in MDS-UPDRS III	-0.34	-0.57,-0.11	Valid	15%; P = 0.32	No serious risk	No serious inconsistency	No serious	Serious	Undetected	Would not	No	No	Moderate
											reduce effect			
2	Change in MDS-UPDRS IV	-0.08	-0.82,0.66	Null	81%;P=0.02	No serious risk	Serious inconsistency	No serious	Serious	NA	Would not	No	No	Low
											reduce effect			
4	Change in Non-motor symptom scale	-0.11	-0.94,0.72	Null	86%;P<0.0001	No serious risk	Serious inconsistency	No serious	No serious	Undetected	Would not	No	No	Moderate
											reduce effect			
2	Change in MMSE	-0.01	-0.30,0.29	Null	0%; P = 0.70	No serious risk	No serious inconsistency	No serious	Serious	NA	Would not	No	No	Moderate
											reduce effect			
3	Change in MoCA	0.04	-0.53,0.60	Null	74%; P = 0.02	No serious risk	Serious inconsistency	No serious	Serious	Undetected	Would not	No	No	Low
											reduce effect			
5	Change in PDQ-39	-0.19	-0.58,0.20	Null	59%; P = 0.05	No serious risk	Serious inconsistency	No serious	Serious	Undetected	Would not	No	No	Low
											reduce effect			
5	Change in BMs	1.27	0.35,2.2	Valid	92%; P<0.00001	No serious risk	Serious inconsistency	No serious	No serious	Undetected	Would not	No	No	Moderate
											reduce effect			
2	Change in Use of laxatives	-0.33	-0.65,-0.02	Valid	0%; P = 0.44	No serious risk	No serious inconsistency	No serious	Serious	NA	Would not	No	No	Moderate
											reduce effect			
2	Change in TAC	0.29	-0.04,0.62	Null	0%; P = 0.98	No serious risk	No serious inconsistency	No serious	Serious	NA	Would not	No	No	Moderate
											reduce effect			
2	Change in GSH	0.51	-0.02,1.03	Null	57%; P = 0.13	No serious risk	Serious inconsistency	No serious	Serious	NA	Would not	No	No	Low
											reduce effect			
2	Change in MDA	-0.69	-1.23,-0.15	Valid	58%; P = 0.12	No serious risk	Serious inconsistency	No serious	Serious	NA	Would not	No	No	Low
											reduce effect			
2	Change in GDS-15	-0.37	-0.87,0.12	Null	0%; P = 0.41	No serious risk	No serious inconsistency	No serious	Serious	NA	Would not	No	No	Moderate
											reduce effect			

SMD, Standard Mean Difference; 95%CI, 95% Confidence Interval; I^2,^ I squared, GRADE, Grading of Recommendations Assessment, Development and Evaluation, MDS-UPDRS, Movement Disorders Society-Unified Parkinson's Disease Rating Scale, MMSE, Mini-Mental State Examination, Moca, Montreal Cognitive Assessment; PDQ-39, Parkinson's Disease Questionnaire-39; BMs, Bowel Movements, TAC, Plasma total antioxidant capacity, GSH, total glutathione, MDA: malondialdehyde; GDS-15, Geriatric Depression Scale-15, NA, Not applicable

## 4 Discussion

Clinical trials revealed that probiotics can alleviate gastrointestinal issues in PD patients (Tan et al., 2021). A study by Cryan et al. suggested that probiotics may reduce neuroinflammation induced by GM and promote the survival of DNs (Cryan et al., 2019). Additionally, an RCT by Tamtaji et al. (2019) demonstrated that probiotics containing bifidobacteria and lactobacilli significantly reduced MDS-UPDRS scores in PD patients, indicating their potential to improve motor symptoms. Probiotics can alleviate both motor and non-motor symptoms by regulating the vagus nerve and gut hormone secretion, promoting the synthesis of neurotransmitters such as serotonin and dopamine (Zhou et al., 2019).

Furthermore, probiotics effectively alleviate such non-motor symptoms as constipation and anxiety (Tamtaji et al., 2019) by balancing GM, reducing intestinal inflammation, and improving intestinal barrier function (Mertsalmi et al., 2017). A study suggested that probiotics can improve sleep quality in PD patients. Sun et al. (2018) found in an animal test that probiotics can modulate GM metabolism by increasing the production of SCFAs, thereby raising levels of sleep-related neurotransmitters, such as serotonin (Sun et al., 2018). Moreover, the immunoregulatory function of probiotics in multiple sclerosis (MS) has been confirmed. A study found that probiotics can improve MS pathology by decreasing pro-inflammatory cytokines, which include TNF-α and IL-1β (Tan et al., 2021).

This study appraised the efficacy and safety of GM-targeted therapies in PD, aiming to provide insights for clinical treatment. GM-targeted therapies can significantly improve MDS-UPDRS I, MDS-UPDRS III, NMSS, PDQ-39, BMs, use of laxatives, and MDA, but had no clear effect on MDS-UPDRS II, MDS-UPDRS IV, MMSE, MoCA, TAC, GSH, or GDS-15. Considering this factor, we degraded the corresponding evidence in the GRADE analysis to provide more objective findings. Wu et al. conducted a meta-analysis on the efficacy and safety of probiotics in treating mild cognitive impairment and Alzheimer’s disease. It revealed that probiotics outperformed the control group in improving the MMSE scores, with the difference between the two groups being statistically significant. The difference in the incidence of adverse events between the two groups was not statistically significant. Similarly, the subgroup analysis revealed no statistically significant difference in MMSE scores between the single-strain probiotics group and the control group, while the composite probiotics outperformed the control group. In this study, we not only analyzed MMSE and MoCA but also examined PD-related motor and non-motor symptoms, filling data gaps from previous studies. However, our study found no significant difference in the effects of probiotics-targeted therapies on MMSE, possibly due to variations in the number of literature. Numerous studies demonstrated no significant differences in the incidence and type of adverse events between the FMT group and the control group. FMT treatment typically lasts 24 to 48 hours. Common gastrointestinal symptoms include discomfort, bloating, diarrhea, constipation, and fever. These symptoms were mild and self-limiting, usually resolving spontaneously within two days (Paramsothy et al., 2017). Despite probiotics being generally considered safe, their long-term risks remain unclear and require validation in large-scale, long-term follow-up studies.

A study by Hill-Burns et al. (2017) demonstrated significant changes in GM composition in PD patients, with beneficial bacteria (such as lactobacilli and bifidobacteria) reduced and potentially pathogenic bacteria (such as Enterobacteriaceae and Prevotella) greatly increased (Hill-Burns et al., 2017). This dysbiosis may impact PD pathogenesis via two mechanisms. Firstly, the initiation of neuroinflammation contributes to PD pathogenesis. The key pathological hallmarks of PD are neuroinflammation and the loss of midbrain dopamine neurons in the nigrostriatal pathway. Lipopolysaccharide (LPS) is considered a key trigger for neuroinflammation (Keshavarzian et al., 2015). Studies indicated that LPS exacerbated neurodegeneration by activating microglia and releasing pro-inflammatory cytokines, which included TNF-α and IL-6. Sun et al. (2018) found substantially reduced levels of SCFAs in PD patients. This reduction may compromise gut barrier integrity, allowing toxic metabolites to pass through the blood-brain barrier and aggravate brain inflammation. An animal test conducted by Sun et al. (2018) showed that FMT could restore SCFAs levels and reduce inflammation, thereby greatly improving motor function (Sun et al., 2018). Secondly, gut barrier dysfunction is considered a potential pathogenic mechanism of PD. In PD patients, compromised gut barrier integrity may lead to the misfolding of the α-synuclein protein. The increased intestinal permeability is mainly caused by α-syn expression, the presence of E. coli, and elevated levels of lipopolysaccharide-binding protein (Forsyth et al., 2011). The supplementation of probiotics and prebiotics can help restore gut barrier function.

This study has several limitations. A total of 11 RCTs were included, with sample sizes ranging from 11 to 128 participants, which may cause small sample size effects, thereby limiting the generalizability of the findings. Future studies can increase sample sizes or conduct multi-center studies to improve quality. Significant heterogeneity was observed in this study for the following outcomes: change in MDS-UPDRS I, change in MDS-UPDRS II, change in MDS-UPDRS IV, change in NMSS, change in MoCA, change in PDQ-39, change in BMs, change in GSH, and change in MDA. Although substantial heterogeneity was observed in several analyses, the limited number of studies included for each outcome precluded reliable subgroup analyses (such as by intervention type, treatment duration, or disease severity) to explore sources of heterogeneity. With the publication of additional studies in the future, subgroup analyses or meta-regression may allow a more in-depth investigation of these sources. However, the findings were objectively interpreted by considering heterogeneity in the GRADE analysis. Publication bias was observed in changes in BMs, potentially due to the small sample size and regional bias. Most studies were conducted in Europe and America, with fewer from Asia and none from other regions such as North America, Africa, or South America. This may introduce regional bias and limit the applicability of the findings to populations with different diets or genetic backgrounds. Future research should encourage more multi-center collaborations on a global scale.

Since regional bias may contribute to publication bias, more multicenter and international studies involving greater sample sizes are required to reduce publication bias. Although most adverse events reported in this meta-analysis were mild (mainly gastrointestinal symptoms such as bloating and diarrhea) and were generally self-limiting, it should be noted that the majority of included studies had short follow-up periods and lacked long-term safety data. Caution is still warranted regarding prolonged use of probiotics, the long-term risks of FMT (such as potential infection transmission or immune effects), and complex metabolic impacts from certain strains. Future large-scale, long-term RCTs should prioritize the assessment of the long-term safety and tolerability of these interventions.

Furthermore, the functional consequences of these microbial shifts are complex and not fully understood. For instance, while some species of Lactobacillus are commonly used as probiotics and considered beneficial, certain strains have been shown to possess decarboxylase activity capable of metabolizing exogenous levodopa (Maini Rekdal et al., 2019). This may potentially reduce the bioavailability of the primary medication for PD, highlighting that the effects of gut microbiota on PD are not straightforward and may be strain-specific and context-dependent. Therefore, simplistic categorization of bacteria as “beneficial” or “pathogenic” in PD is misleading. Future research and therapies should move beyond taxonomic associations to focus on specific bacterial functions, host-microbe interactions, and their net effect on drug metabolism and disease progression.

## 5 Conclusion

In this study, GM-targeted therapies were found to significantly improve MDS-UPDRS III, BMs, use of laxatives, and MDA in PD patients. According to the GRADE assessment, change in MDS-UPDRS I, change in MDS-UPDRS III, change in NMSS, change in MMSE, change in BMs, change in use of laxatives, change in TAC, and change in GDS-15 (PSA response rate) were rated as moderate evidence. Given the limitations of small sample sizes, potential heterogeneity, and publication bias, future studies should involve larger, multi-center RCTs to further evaluate the long-term efficacy and safety of GM-targeted therapies in patients with PD.

## Data Availability

The original contributions presented in the study are included in the article/supplementary material. Further inquiries can be directed to the corresponding author.
